# Capsaicin: Friend or Foe in Skin Cancer and Other Related Malignancies?

**DOI:** 10.3390/nu9121365

**Published:** 2017-12-16

**Authors:** Simona-Roxana Georgescu, Maria-Isabela Sârbu, Clara Matei, Mihaela Adriana Ilie, Constantin Caruntu, Carolina Constantin, Monica Neagu, Mircea Tampa

**Affiliations:** 1 Department of Dermatology, Carol DavilaUniversity of Medicine and Pharmacy, 020021 Bucharest, Romania; simonaroxanageorgescu@yahoo.com (S.-R.G.); isabela_sarbu@yahoo.com (M.-I.S.); matei_clara@yahoo.com (C.M.); tampa_mircea@yahoo.com (M.T.); 2 Department of Biochemistry, Carol Davila University of Medicine and Pharmacy, 020021 Bucharest, Romania; mihaelaadriana2005@yahoo.com; 3 Department of Physiology, Carol Davila University of Medicine and Pharmacy, 050474 Bucharest, Romania; 4 Department of Dermatology, Prof. N.C. Paulescu National Institute of Diabetes, Nutrition and Metabolic Diseases, 011233 Bucharest, Romania; 5 Immunology Department, Victor Babes National Institute of Pathology, 050096 Bucharest, Romania; caroconstantin@gmail.com (C.C.); neagu.monica@gmail.com (M.N.); 6 Faculty of Biology, University of Bucharest, 76201 Bucharest, Romania

**Keywords:** capsaicin, skin, neurogenic inflammation, cancer, carcinogenesis, squamous cell carcinoma, melanoma

## Abstract

Capsaicin is the main pungent in chili peppers, one of the most commonly used spices in the world; its analgesic and anti-inflammatory properties have been proven in various cultures for centuries. It is a lipophilic substance belonging to the class of vanilloids and an agonist of the transient receptor potential vanilloid 1 receptor. Taking into consideration the complex neuro-immune impact of capsaicin and the potential link between inflammation and carcinogenesis, the effect of capsaicin on muco-cutaneous cancer has aroused a growing interest. The aim of this review is to look over the most recent data regarding the connection between capsaicin and muco-cutaneous cancers, with emphasis on melanoma and muco-cutaneous squamous cell carcinoma.

## 1. Introduction

Chili peppers belong to the genus *Capsicum* of the *Solanaceae* family and are some of the most used condiments in the world being consumed on daily basis by almost 25% of the population [1,2,3,4,5,6]. The chili extract has been long used in traditional medicine. Alcoholic hot pepper extract was used as a counterirritant analgesic and helped treat burning sensations and pruritus. In tropical countries it was administrated to induce vasodilatation and to increase heat loss [7]. 

The main pungent component in chili peppers is capsaicin and this plant component is probably produced as a defense mechanism against herbivores and fungi [6]. Capsaicin, an alkylamide, is the most abundant capsaicinoid found in chili peppers (69%) but dihydrocapsaicin (22%), nordihydrocapsaicin (7%), homocapsaicin (1%) and homodihydrocapsaicin (1%) are also present [1]. The history of capsaicin goes back to the 19thcentury. In 1816, Bucholtz managed for the first time the extraction as a solution of the pungent component from the chili pepper [8]. In 1846, Thresh named this component capsaicin and achieved for the first time its isolation in pure, crystalline form [9]. Another important moment is the identification of the exact structure of capsaicin, which was communicated in 1919 by Nelson [10]. There are still recent studies that try to improve the isolation and purification of capsaicin from the capsaicinoid extract [11] reinforced by studies that reveal that there are clear regulations of the composition during fruit ripening [12]. In 1930, Späth and Darling synthesized capsaicin for the first time [13]. The 20th century has thus established capsaicin as a compound with various actions besides being a natural food additive [14,15].

## 2. Capsaicin and Neurogenic Inflammation

Capsaicin (*trans*-8-methyl-N-vanillyl-6-noneamide) is a lipophilic substance, belonging to the class of vanilloids [16]; its molecular formula is C_18_H_27_NO_3_ and its molecular weight is 305.4 Da. Capsaicin is an agonist of the transient receptor potential vanilloid 1 receptor (TRPV1) which is a member of the transient receptor potential (TRP) family of cation channels [17]. 

Besides capsaicin, TRPV1 can be activated by temperatures of 43 °C or higher, by acidity (pH<6), endocanabinoids such as anandamide, metabolites of polyunsaturated fatty acids or other vanilloids [18]. Its function can also be modulated by inflammatory mediators, such as bradykinin and prostaglandin E2 with a facilitatory effect induced probably by protein kinases (PKC or PKA) -mediated receptor phosphorylation [19,20,21]. Other agents like nerve growth factor (NGF), catecholamines, histamine can also increase TRPV1 responses [22,23,24]. 

TRPV1 receptors are expressed in the central nervous system and in sensory neurons of the dorsal root ganglion, but also in non-neuronal tissues [25]. In the skin, TRPV1 is present in the unmyelinated type C and thin myelinated A-delta sensory nerve fibres, keratinocytes, mast cells, dermal blood vessels, fibroblasts, hair follicles, vascular smooth muscle cells, sebocytes and eccrine sweat glands [26,27,28]. TRPV1 might therefore play the role of extraneuronal receptor [29]. To date, it has been suggested that TRPV1 might play a role in mastocyte activation [30], release of proinflammatory mediators from keratinocytes [31] and modulation of proliferation, differentiation and apoptosis of keratinocytes from the outer root sheath [32]. 

**Figure 1:**
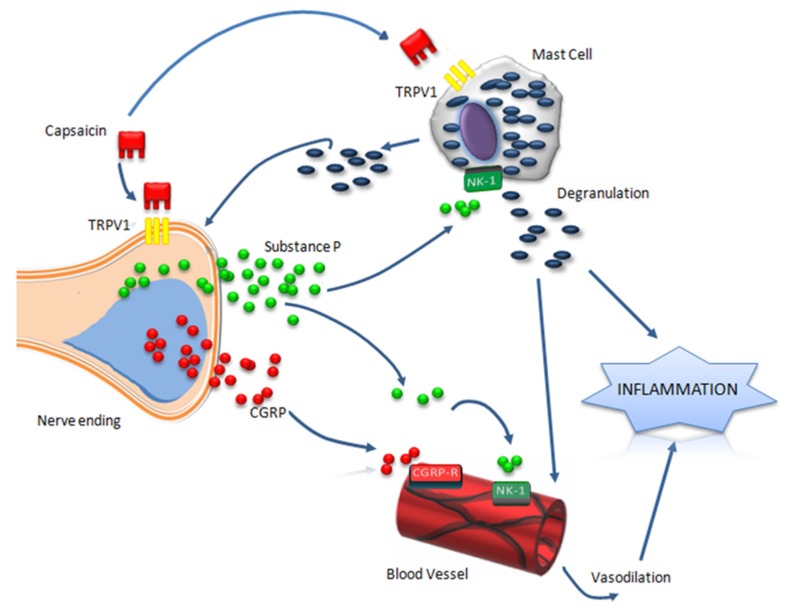
Capsaicin-induced inflammatory response is initiated by activation of transient receptor potential vanilloid 1 receptor(TRPV1) followed by the release of pro-inflammatory neuropeptides from nerve endings. Substance P(SP)and calcitonin-gene related peptide(CGRP), by activation of neurokinin-1 receptor (NK-1) and CGRP receptors, induce vasodilation, increased vascular permeability and release of pro-inflammatory cytokines. The released neuropeptides can induce degranulation of mast cells that play an important role in amplification of capsaicin-induced neurogenic inflammation.

Applied on the skin or oral mucosa, capsaicin induces initially a local burning sensation [26], followed by allodynia and hyperesthesia to mechanical and heat stimulation [33]. These nociceptive effects are associated with a transient local wheal and flare response known as neurogenic inflammation, triggered by the release of neuropeptides from the cutaneous sensory nerve endings (see Figure 1) [34,35]. Substance P (SP) and calcitonin-gene related peptide (CGRP) are recognized as the most important neuropeptides within neurogenic inflammation [36]. SP acts upon micro vascularization through its neurokinin-1 receptor (NK-1R) and has vasodilatory effects, increases vascular permeability and favors the release of pro-inflammatory cytokines [37], whilst CGRP induces microvascular dilatation resulting in increased blood flow [38]. Besides the neuropeptides release from nerve fibers, activation of mast cells has an important role in the capsaicin-induced inflammatory reaction [39]. Neuropeptides, with SP having the most significant effects, induce mast cell degranulation and synthesis of pro-inflammatory cytokines [40,41]. Mast cell mediators in turn activate nociceptors and further amplify the release of neuropeptides from the sensory nerves [39].

On the other hand, capsaicin blocks the axoplasmic transport of substance P and somatostatin in sensory neurons, thus depleting the neuropeptides [6,42,43] and progressively reducing the initial local inflammatory effect, explaining the potential use of capsaicin in the treatment of chronic inflammatory skin diseases [28].

Moreover, subsequent applications of capsaicin lead to desensitization which is responsible for the analgesic effect of topical capsaicin [6,44] and its wide use in the treatment of neuropathic pain [45], post-herpetic neuralgia, diabetic neuropathy, post-surgical neuralgia, post-traumatic neuropathy and musculoskeletal pain [6,46]. 

Capsaicin can also have neurotoxic effects and can induce a gradual degeneration of cutaneous nerve fibers when used in high concentrations or for a long period of time [47,48,49]. 

Thus, capsaicin, depending on the duration and intensity of stimulation, can induce opposite effects, and the study of capsaicin-induced reactions has aroused the interest of both researchers and clinicians from a broad range of specialties.

## 3. Capsaicin and Cancer

Various studies have suggested a potential pro-carcinogenic role of capsaicin use [3] further supported by the potential connection between inflammation and tumorigenesis. In some cases, pro-inflammatory cytokines/chemokines can trigger malignant transformation and tumor associated inflammation in turn can promote proliferation and survival of malignant cells [50,51]. 

However, other recent studies indicate more to a protective effect against various types of cancer via different pathways, mostly unrelated to TRPV1 [3,52,53,54,55,56,57,58,59,60]. Thus, we will elaborate further on the capsaicin involvement in muco-cutaneous squamous cell carcinoma and melanoma, as the main malignancies where capsaicin has proven its involvement (see Table 1).

### 3.1. The Impact of Capsaicin on Muco-Cutaneous Squamous Cell Carcinoma

Muco-cutaneous squamous cell carcinoma is one of the most frequent malignancies among Caucasians and its incidence has increased in the last decades, probably due to lifestyle changes and the increased proportion of aged populations [76,77,78,79]. Muco-cutaneous squamous cell carcinoma is responsible for most deaths associated with non-melanoma muco-cutaneous cancer. It may generate major defects both aesthetically and functionally and require a complex therapeutic approach, depending on the stage of the disease and the general status of the patient [76,77,78,79,80,81]. For that reason, muco-cutaneous squamous cell carcinoma is an important public health problem and new therapeutic approaches are necessary [82,83,84,85,86,87,88,89,90].

The most important risk factors for the development of muco-cutaneous squamous cell carcinoma are fair skin type, chronic exposure to ultraviolet radiation (UVR), exposure to ionizing radiation, smoking, exposure to chemical carcinogens, human papillomavirus (HPV) infections and genetic predisposition [80,81,87,88,89,91,92,93]. 

Moreover, various studies have shown that neuroendocrine factors might play a role in the development of muco-cutaneous squamous cell carcinoma [94]. The release of CGRP and substance P, as well as other neuropeptides, from unmyelinated c-fibres and myelinated A delta-fibres of sensory nerves, a well-known effect triggered by capsaicin is also induced by UVR exposure and may contribute to induction of carcinogenesis [94,95]. CGRP has important vasodilatory effects on small and large vessels, potentiates microvascular permeability and edema caused by SP, enhances in vitro keratinocyte and melanocyte proliferation and is a potent immunomodulator [94,95,96,97]. By impairing the function of cutaneous macrophages and Langerhans cells, CGRP is a potent inhibitor of acute and delayed type hypersensitivity reactions [95] but also interferes with anti-tumoral immune response initiation [94]. 

SP is a member of the tachykinin family which has vasodilatory effects, induces protein extravasation, lymphocyte proliferation, chemotaxis, activates macrophages and promotes the secretion of interleukin 1 (IL-1), IL-6and TNF-α [94,95,98]. It has been associated with stress induced mast cell activation [41]. The effects of SP are mediated through NK-1R, which is widely expressed in the brain, skin, intestine, lung and immune cells [94,95]. There is some evidence that SP and NK1-R might be involved in the development and progression of cancer. Thus, SP has been associated with cell proliferation and migration in esophageal squamous cell carcinoma (SCC) [99], melanoma [100,101], retinoblastoma [102], neuroblastoma and glioma [103]. Brener et al. investigated the presence of SP and NK-1R in 93 oral SCC from 73 patients and concluded that the SP/NK-1R system might have a role in tumor development and progression [104]. Other authors studied the distribution of SP and NK-1R in esophageal SCC and found a higher density of SP positive nerve fibres and NK-1R expression in carcinoma cells, thus concluding that SP and NK-1R promote growth and migration of esophageal SCC cells [99]. Considering the evidence regarding the role of SP in the development of the disease some authors suggested that NK-1R antagonists might be useful in the treatment of oral cancer [104].

Taking into consideration the complex neuro-immune impact of capsaicin and the potential link between inflammation and carcinogenesis, the effect of capsaicin on muco-cutaneous cancer has aroused a growing interest. Since several reports indicated that the consumption of chili peppers might be associated with an increased risk of cancer [105], some authors studied the effect of long term capsaicin treatment. Toth and Gannett found that, after a lifelong diet with capsaicin, 22% of female mice and 14% of male mice had tumors of the cecum. In the control group only 8% of mice had cecum tumors [106]. Chanda et al. assessed the oncogenic potential of topical trans-capsaicin applied for 26 weeks in Tg.AC mice. The Tg.AC mice received trans-capsaicin dissolved in diethylene glycol monoethyl ether (DGME). Mice from the positive control group received tetradecanoylphorbol-13-acetate (TPA) dissolved in DGME and controls received lidocaine. The authors found that topical capsaicin was not associated with an increased incidence of preneoplastic and neoplastic lesions as compared to the concurrent vehicle or lidocaine while the TPA treated mice had multiple skin papillomas. The authors therefore concluded that trans-capsaicin, lidocaine and DGME should be considered non-oncogenic [107].

Le et al. studied the effect of capsaicin on human pharyngeal SCC cells (FaDu) and found that capsaicin inhibits growth and proliferation in a time and dose dependent manner and induces apoptosis via mitochondrial pathways [61]. The authors also analyzed the expression of the anti-apoptotic Bcl-2 gene and the pro-apoptotic Bax and Bad genes and found a reduction of Bcl-2 gene and enhanced expression of Bax and Bad genes [61]. Gonzales et al. studied the anti-tumor effect of capsaicin, a TRPV1 agonist, and capsazepine, a TRPV1 antagonist, on oral squamous cell carcinoma (OSCC) cell lines; the authors found that capsaicin alone reduced cell viability [62]. The association of capsazepine and capsaicin not only did not reverse the effect of capsaicin but capsazepine alone was also cytotoxic to tumor cells; the authors therefore concluded that the antiapoptotic effect of vanilloids is independent of TRPV1 and suggested that the induction of reactive oxygen species is responsible for apoptosis [62].

Han et al. showed in a study published in 2002 that topical application of capsaicin on the skin of female ICR mice suppresses phorbol ester-induced activation of nuclear factor kappaB (NF-κB) and activator protein 1 (AP-1) and concluded that this might be responsible for the chemopreventive effects of capsaicin [63]. These results are congruent with findings previously reported by another group of authors [108,109,110,111]. Surh et al. studied the chemoprotective effect of capsaicin against tumorigenesis and mutagenesis produced by vinyl carbamate (VC) and *N*-nitrosodimethylamine(NDMA) also on female ICR mice [64]. The authors found that topical capsaicin pre-treatment lowered the number of VC-induced tumors by 60% and hypothesized that capsaicin suppresses tumorigenesis and mutagenesis by inhibiting cytochrome P-450 IIE1 isoform [64].

### 3.2. The Effect of Capsaicin on Melanoma

Melanoma is a malignant tumor that arises from melanocytes; melanocytes are melanin-producing cells situated in the basal layer of epidermis, in uveal structures of the eye and in the meninges; of all possible sites, skin is the most frequent location of melanoma [87,88]. Even though melanoma is less frequent than most malignant cutaneous tumors (i.e., basal cell carcinoma, squamous cell carcinoma), it has the most aggressive course, accounting for more than 75% of all skin cancer deaths. Melanoma can occur at any age but it is more frequent between 30 and 70 years; females are more frequently affected than males (male:female ratio 1:1.5) [88,89]. The incidence of melanoma has been on the rise worldwide in the last decades. Excessive ultraviolet radiation exposure (from both sun and artificial sources—e.g., tanning beds) especially under the age of 20, skin phototypes I and II (light skin pigmentation), genetic predisposition, increased number of melanocytic nevi and the presence of atypical nevi are the main risk factors for developing melanoma. Most melanomas occur de novo [90,112,113]. 

The treatment of melanoma varies depending on the stage of the disease. Surgical excision is the mainstay treatment for primary melanoma. Metastatic melanomas however require chemotherapy, immunotherapy or palliative treatment. These are usually associated with severe adverse reactions and low response rates [87,88]. Therefore, new drugs as well as new ways of investigating their efficacy have been elaborated [114]. The prognosis of patients with metastatic melanoma was improved after the introduction of BRAF(B-Rafenzyme) inhibitors (vemurafenib, dabrafenib), mitogen-activated protein/extracellular signal-regulated kinase kinase(MEK) inhibitors (trametinib) and immune checkpoint inhibitors (nivolumab, ipilimumab) [113,115].These therapies however are very expensive and are not available for all the patients [113,115].

Under these circumstances, there is a real need to identify new therapeutic targets in order to develop cheaper, but efficient, treatment options. Hence, the mechanisms behind the development and progression of melanoma were intensely studied and recent reports showed that neuro-endocrine factors might be involved [100,101,116,117]. Several studies have investigated the potential role of NK-1R and SP, one of the main neuropeptides involved in capsaicin-induced inflammatory reaction. A recent study performed on canine melanoma tissues and cell lines found that 11 of 15 tumors revealed NK-1R immunoreactivity [118]. The expression of SP in malignant melanoma and melanoma precursors was also studied and the authors showed that 68% of primary invasive melanomas, 40% of metastatic melanomas, 60% of in situ melanomas and 58% of dysplastic nevi express the neuropeptide [119]. SP and NK-1R are also involved in melanogenesis [120]. B16-F10 melanoma cells treatment with SP results in activation of NK-1R, phosphorylation of p70 S6K1, inhibition of p38mitogen-activated protein kinase(MAPK), down-regulation in tyrosinase activity and suppression of melanogenesis [121]. There is increasing evidence regarding the involvement of SP and NK-1R in melanoma cells proliferation [100,101,122,123]. For that reason, NK-1R is now regarded as a target in melanoma treatment and NK-1R antagonists are being intensely studied [100,101,122,123].

The direct role of capsaicin in the treatment of melanoma was investigated in several studies, as explained further [65,66,67,68,69,70,71,72,124,125,126,127,128,129]. Morré et al. studied the effect of capsaicin on nicotinamide adenine dinucleotide(NADH) oxidase activity of plasma membranes and cell growth of human primary melanocytes and melanoma cells (A-375 and SK-MEL-28 cell cultures) [65]. The authors found that capsaicin inhibits plasma membrane NADH oxidase activity preferentially in melanoma cells thus inhibiting growth and increasing apoptosis [65]. Brar et al. also showed in a study performed on human melanoma cell lines that reactive oxygen species produced endogenously from nicotinamide adenine dinucleotide phosphate-reduced(NAD(P)H):quinone oxidoreductase activate NF-κB in melanoma cells in an autocrine fashion and that capsaicin significantly reduces proliferation of melanoma cells [66].

Patel et al. showed in a study published in 2002 that the NF-κB activation regulates the expression of IL-8 in melanoma cells and that the addition of capsaicin determines the inhibition of constitutive and IL1-beta and TNF-α induced IL-8 expression in melanoma cells [67]. In melanoma, IL-8 over-expression is associated with the transition from radial growth phase to vertical growth phase and with the development of metastases [124,125]. Capsaicin is a potent inhibitor of NF-κB. It suppresses the activation of NF-κB by inhibiting IκBα (nuclear factor of kappa light polypeptide gene enhancer in B-cells inhibitor, alpha) degradation and blocking the translocation of p65 in human promyelocytic leukaemia HL-60 cells [63,126].

In a study published in 2012, Kim aimed to explain the mechanism by which capsaicin induces apoptosis in melanoma cells [68]. The author therefore studied the role of nitric oxide (NO) during apoptosis induced by capsaicin and resveratrol on A375 human melanoma cells and found that NO stimulates p53 and induces conformational changes in Bax and Bcl-2 and activates caspases 3, 8 and 9. The authors concluded that capsaicin and resveratrol activate the mitochondrial and death receptor pathways [68]. 

In a study published in 2008, Shin et al. evaluated the effects of capsaicin on highly metastatic B16-F10 mouse melanoma cells and found that capsaicin inhibits migration of melanoma cells in a dose-dependent manner [69]. The authors also found that capsaicin decreases the phosphorylation of the p85 regulatory subunit of phosphatidylinositol 3-kinase (PI3-K) and Akt and concluded that capsaicin down-regulates the PI3-K/Akt pathway. Furthermore, the authors found that capsaicin inhibits the Rac1 activity [69]. The PI3-K/Akt pathway is one of the main signaling networks in cancer and plays an important role in melanoma initiation and in therapeutic resistance [127,128]. Rac1 is involved in cell migration and metastasis [129]. Jun et al. also studied the effect of capsaicin on B16-F10 murine melanoma cells. The authors found that capsaicin determines release of mitochondrial cytochrome c, activation of caspase-3 and cleavage of poly (ADP-ribose) polymerase and finally induces apoptosis of melanoma cells through down regulation of Bcl-2 [60]. Other studies have observed induction of apoptosis by capsaicin in melanoma cells, as well: Gong et al. showed, in a study performed on human melanoma A375-S2 cells, that capsaicin induces melanoma cell death in a time and dose dependent manner by reducing the expression of inhibitor of caspase activated DNase (ICAD); ICAD expression was decreased over the lapse of time, as cell treated with capsaicin progressed into apoptotic stages [70]. Some authors studied the combined effect of capsaicin and other agents on melanoma cells [71,72]. Marques et al. investigated the apoptotic effect of capsaicin and HA14-1, a small molecular compound that inhibits the anti-apoptotic effect of Bcl-2, on melanoma cells, melanocytes and fibroblasts [71]. The authors found that capsaicin induces apoptosis in melanocytes and HBL, A375SM and C8161 melanoma cell lines at lower concentrations than in fibroblasts and that the capsaicin and HA14-1 combination shows additive inhibitory effect on melanoma and melanocyte viability, inducing apoptosis in two of the three studied melanoma cell lines [71]. The authors concluded that capsaicin can be associated with other organic compounds as a pro-apoptotic agent to reduce toxicity and adverse reactions [71]. Schwartz et al. studied the combined effect of hydroxycitrate, lipoic acid and capsaicin on lung cancer cells, bladder cancer cells and melanoma cells and found that the association of these drugs is effective in inducing tumor regression and lacks toxicity [72].

Taking into account the increasing evidence regarding its anti-carcinogenic role, expanding the research on capsaicin actions may lead to identification of potential new therapeutic pathways.

### 3.3. Capsaicin’s Involvement in Carcinogenesis

A potential co-carcinogenic role of capsaicin has aroused the interest of various researchers. A study published in 2009, showed that TRPV1 interacts with the epidermal growth factor receptor (EGFR) and determines its degradation though the lysosomal pathway [73]. EGFR is a receptor tyrosine kinase with an important role in the development of the epidermis, which is overexpressed in many epithelial cancers. Using a skin carcinogenesis model with 7,12-dimethyl benz(a)anthracene (DMBA) and TPA in TRPV1^−/−^ (knockout) and TRPV1^+/+^ (wild type) mice, authors have shown that TRPV1^−/−^ mice developed significantly more skin tumors than TRPV1^+/+^ mice [73]. Moreover, to assess to role of EGFR in skin carcinogenesis, the authors performed the same experiment, except that some of the mice received an EGFR inhibitor; the scientists discovered that carcinogenesis was substantially more suppressed in TRPV1^−/−^ mice, after EGFR inhibitor was administered [73]. 

Another study published in 2010 showed that topical application of capsaicin on the skin of TRPV1 wildtype mice and TRPV1 knockout mice, which were previously subjected to the two-stage skin carcinogenesis experiment with DMBA (9,10-Dimethyl-1,2-benzanthracene) and TPA, was associated with significantly more and larger tumors than TPA treatment alone [25]. TRPV1 knockout mice were more affected than TRPV1 wildtype mice. Mice treated with capsaicin alone however have not developed any tumors. These findings suggest that carcinogenesis has a TRPV1 independent mechanism. Further research revealed higher levels of COX-2(cyclo-oxygenase-2) in mice treated with capsaicin and TPA than in mice treated with TPA alone thus suggesting that capsaicin induces an increased COX-2 expression in the presence of TPA. COX-2 expression was increased in EGFR wildtype cells but not in EGFR knockout cells. The authors therefore suggest that capsaicin acts as a co-carcinogen through EGFR dependent mechanisms/pathways [25].

The link between capsaicin receptor and skin tumorigenesis was the subject of an experimental in vivo research which found that topical application of TRPV1-antagonist AMG9810[(E)-3-(4-t-Butylphenyl)-N-(2,3-dihydrobenzo[b][1,4] dioxin-6-yl)acrylamide]promotes tumor development in mice previously treated with DMBA. The levels of EGFR were also higher in these mice as compared to the control group. Moreover, the phosphorylation level of EGFR was significantly increased in AMG9810 treated mice compared to the control groups. EGFR phosphorylation activates the Akt/mTOR-signaling pathway which has an important role in tumorigenesis. It was therefore concluded that the TRPV1 antagonist induces carcinogenesis by activating the EGFR/Akt/mTOR signaling pathway [74].

Liu et al. also studied the effect of topical applications of capsaicin on the dorsal skin of mice in which carcinogenesis was induced by DMBA/TPA. The authors showed that capsaicin led to the appearance of more numerous and larger skin tumors as compared to the control group and suggested that Erk, p38 and inflammation may play an important role in the cancer-promoting effect of capsaicin [75].

All these findings suggest that, even though capsaicin itself is not a carcinogen, long-term application of capsaicin for pain relief might increase the risk of carcinogenesis when it is associated with a tumor promoter [73].

## 4. Conclusions

Capsaicin is one of the most commonly used spices in the world and its analgesic and anti-inflammatory properties have been known for centuries. Short term administration of capsaicin has the ability to trigger the release of neuropeptides like SP and CGRP which might play a role in tumorigenesis. However, chronic administration of capsaicin progressively reduces the initial inflammatory reaction, leading to desensitization or even to neurotoxic effects, depending on the duration and intensity of applications.

In recent years various studies have focused on the potential impact of capsaicin on tumorigenesis, investigating both the anti-carcinogenic and carcinogenic actions of capsaicin. Data available so far regarding the effect of capsaicin on various types of skin cancers suggests that capsaicin has a chemopreventive role. Sinceseveral authors showed that under certain circumstances capsaicin can have a pro-tumorgenic potential, caution is mandatory when capsaicin is administered in conditions that favor tumorigenesis as it might have a co-carcinogenic effect.

## Figures and Tables

**Table 1:** Summarizing the carcinogenic and anti-carcinogenic effects of capsaicin, the primary pathway through which the effect is occurring, and the experimental model used to demonstrate the effect.

Effect of Capsaicin	Primary Pathway through Which the Effect Is Occurring	Model Used to Demonstrate the Effect	References
Anticarcinogenic	Mitochondrial pathway-dependent apoptosis: ↓Bcl-2 ↑Bax, ↑Bad	human pharyngeal SCC cells (FaDu)	Le et al. [61]
	Induction of reactive oxygen species; apoptosis independent oftransient receptor potential vanilloid 1 receptor (TRPV1)	oral squamous cell carcinoma (OSCC) cell lines	Gonzales et al. [62]
	Nuclear factor kappaB (NF-kB), activator protein 1 (AP-1)	ICR mouse model; humanpromyelocytic leukemiaHL-60 cells	Han et al. [63]
	Inhibition of the cytochrome P-450 IIE1 isoform	ICR mouse model	Surh et al. [64]
	↓nicotinamide adenine dinucleotide (NADH) oxidase activity; ↑apoptosis	A375, SK-MEL-28 human melanoma cell lines; B16 murine melanoma cell line	Morré et al. [65]
	↓nicotinamide adenine dinucleotide phosphate-reduced(NAD(P)H): quinone oxidoreductase ; ↓NF-κB	CRL 1585 and CRL 1619 human melanoma cell lines	Brar et al. [66]
	↓activation of constitutive and IL-1beta-induced NF-κB	Human melanoma cells	Patel et al. [67]
	↑p53, induces apoptosis via Bcl-2, Bax, caspases 3,8,9	A375 human melanoma cell line	Kim [68]
	Down-regulation of PI3-K/Akt pathway	B16-F10 mouse melanoma cells	Shin et al. [69]
	Downregulation of Bcl-2; induction of apoptosis	B16-F10 mouse melanoma cells	Jun et al. [60]
	↓caspase-activated DNase inhibitor(ICAD)expression; induction of apoptosis	human melanoma A375-S2 cell line	Gong et al. [70]
	Induction of apoptosis	melanocytes and HBL, A375SM, C8161 melanoma cell lines	Marques et al. [71]
	Delays tumor growth	melanoma B16-F10; mouse model	Schwartz et al. [72]
Cocarcinogenic	Epidermal growth factor receptor(EGFR) pathway	DMBA/TPA mouse model	Bode et al. [73]
	EGFR pathway; ↑cyclo-oxygenase-2 (COX-2)	DMBA/TPA mouse model	Hwang et al. [25]
	EGFR/Akt/mTOR signaling pathway	DMBA/TPA mouse model	Li et al. [74]
	Erk/p38 signaling pathway	DMBA/TPA mouse model	Liu et al. [75]
